# Gamma irradiation green synthesis of (polyacrylamide/chitosan/silver nanoparticles) hydrogel nanocomposites and their using as antifungal against *Candida albicans* and anti-cancer modulator

**DOI:** 10.1038/s41598-024-74027-x

**Published:** 2024-10-28

**Authors:** Salwa A. Khalil, Ahmed Awadallah-F, Mervat R. Khaffaga, Rasha Mohammad Fathy, Ahmad S. Kodous

**Affiliations:** 1 Radiation Chemistry Department, National Center for Radiation Research and Technology (NCRRT), Egyptian Atomic Energy Authority (EAEA), Cairo, Egypt; 2 Polymer Chemistry Department, National Center for Radiation Research and Technology (NCRRT), Egyptian Atomic Energy Authority (EAEA), Cairo, Egypt; 3 Drug Radiation Research Department, National Center for Radiation Research and Technology (NCRRT), Egyptian Atomic Energy Authority (EAEA), Cairo, Egypt; 4 Radiation Biology Department, National Center for Radiation Research and Technology (NCRRT), Egyptian Atomic Energy Authority (EAEA), Cairo, Egypt

**Keywords:** Hydrogel, Nanocomposite, Gamma irradiation, Antimicrobial, Anti-cancer, Materials science, Structural materials, Composites

## Abstract

Silver nanoparticles-loaded hydrogel nanocomposites are exploited for medicinal and pharmaceutical applications. Hydrogel nanocomposites were prepared from acrylamide (Am), chitosan (CS) and AgNO_3_ utilizing gamma rays. Diverse variables were applied in preparation of silver nanoparticles-laoded hydrogel nanocomposites of (PAm/CS)-AgNPs such as influence of radiation dose and influnece of CS concentration. Diverse techniques were utilized to characterize hydrogel nanocomposites; Fourier transform infrared spectroscopy (FTIR), thermal gravimetric analysis (TGA), X-ray diffraction (XRD), Transmission electron microscopy (TEM), energy dispersive X-ray (EDX) and scanning electron microscopy (SEM). Results confirmed formation of silver nanoparticles-loaded hydrogel nanocomposites of (PAm/CS)-AgNPs. Antifungal activity of (PAm/CS)-AgNPs hydrogel nanocomposites on viability of *C. albicans* was esitmated. Results displayed the efficient microbial inhibition activity of treatment against *C. albicans* compared to control. Furthermore, (PAm/CS)-AgNPs hydrogel nanocomposite against cervical cancer HeLa cell line was investigated. Cytotoxicity of (PAm/CS)-AgNPs hydrogel nanocomposites on prior cancer cell line empolyed to prohibition of cell growth assesssed by MTT test. HeLa cancer cell is treated by (PAm/CS)-AgNPs for 48 h exposed a potential apoptotic activity by noticeable up-regulation of p53 gene expression. Moreover, anticancer activity was investigated by down-regulation of platelet-based growth variable receptor beta (PDGFR-β), Bcl2, Cathepsine, and MMP-2 gene expression. antioxidant activity was investigated and results showed antioxidant activity of (PAm/CS) hydrogel and (PAm/CS)-AgNPs hydrogel nanocomposite are 87.8% and 62.9%, respectively.

## Introduction

Chitosan is commonly known that is a deacetylated chitin that could be divided into absorbable and non-toxic monosaccharides that are not teratogenic ^1–3^. It is one of the most naturally productive biodegradable polymers sort and are utlized in biomedical initiatives. CS has attractive properties such as antibacterial, anti-hemostasis, anti-thrombotic, anti-inflammatory, and wound addressing features, as well as immunomodulation capabilities ^4–6^. Tumors are the greatest cause of death around the world^1^. By 2030, there are anticipated to be 20–30 million new cancer diagnoses and 13–17 million cancer deaths around the world.^7^ Malignant cells expose all of the following hallmarks: uncontrolled cell growth, the ability to invade growth suppressors, resistance to cell death, unlimited replicative potential, the capability to breakthrough and metastasize, the ability to induce angiogenesis, the capability to reprogram their energy metabolism, and the capability to evade immune detection ^8^. Prospective cancers are on the rise everywhere across the body, thanks in large part to genomic changes created by intrinsic and/or external stressors changing cell processes in the regulations. Human cancer is fundamentally caused by genetics, lifestyle choices like smoking and tobacco chewing, certain illnesses, and exposure to various types of radiation and toxins ^9^. According to globocan, the global cancer incidence, mortality, and revalence database ^10^. Cervical cancer is the fourth most common cancer and the fundamental cause of cancer deaths in women in particular. Different therapies target the p53 and PDGFR-β genes for neuroblastoma and cervical cancer ^11^. Hydrogels are well known as three dimensionally interconnected hydrophilic polymers that can take in more amount of water molecules through their matrices and keeping their structure without dissolving in aqueous media. Because the physicochemical features of hydrogels may be greatly similar to those noticed in biological organs. A result, there is a great deal of effort being put into utilizing hydrogels for biological fields, such as drug delivery system ^12,13^, dressing for wound/burn^14,15^, of tissue engineering scaffolds^16,17^, and others ^18,19^. In specific, the uploading of drugs into hydrogel matrices may show an innovative drug delivery technique. The capability to control the rate of drug release from hydrogel matrices is one of the key advantages of this drug delivery system. This is because hydrogels are responsive to a numerous external stimuli parameters, involving temperature, pH, light, and electric and magnetic fields ^20,21^. Overall, gamma irradiation is a promising technique for synthesizing hydrogel nanocomposites for drug delivery system. It has many advantages over conventional methods, including rapid and facile preparation, non-contaminating preparation and the capability to create dimensional polymeric networks, capability to generate nanomaterials in solution, and capability to sterilize the hydrogel nanocomposites in one technological approach ^22^. In numerous cases, the change in pH value^23^, media temperature^24^, and exposure to light has induced the absorbing actionr, reduction, or hydrogels decomposition to release drug molecules in a given approach. In the absence of external stimuli paramaters, the gradual release of medicine from a hydrogel nanocomposites can guarantee a stable delivery and long-lasting period of drug components. This is because hydrogels are capable to retain a large quantity of water, which lets the drug molecules to be released gradually over time^25^. The utilization of hydrogels to deliver AgNPs is a promising method to enhance the efficacy and safety of antimicrobial therapy. AgNPs containing hydrogels have the potentiality to decrease the need for conventional antibiotics, which can help to decrease the risk of antibiotic resistance ^26^. When AgNPs adheres to bacteria, they release Ag^+^ ions via an oxidation process. Ag^+^ ions are highly reactive and can bind to a variety of molecules in the bacterial cell membrane. One of the important targets of Ag^+^ ions is the bacterial cell membrane. Ag^+^ ions can disrupt the integrity of bacterial cell membrane, causing leakage of intracellular contents and employing to cell death ^27,28^. Moreover, AgNPs have antimicrobial properties; although, they can increase the inflammations of skin ^29^. Thus, it is strongly recommended to separate AgNPs from contaminated site, whilst feeding Ag^+^ cations to a targeted site. In addition, hydrogel involving AgNPs may face the matters; the cross-linked networks of hydrogel can prohibit the releasing of AgNPs from the matrix networks, while leaving the releasing of Ag^+^ cations that are created by AgNPs oxidation into the hydrogel networks. To manufacture AgNPs-based hydrogel nanocomposites, one can use in-situ and ex-situ approach for inducing NPs into hydrogel bodies. In the in-situ approach for preparing AgNP-containing hydrogels, hydrogels are first prepared. Then, Ag^+^ cations are complex with specific chemical functional groups in hydrogel components. Finally, Ag^+^ cations are decreased to form AgNPs ^30,31^. Since the binding of Ag^+^ cations to hydrogel structure conduct in a solution status followed by reduction of Ag^+^ loaded hydrogel by chemical species make sure the homogeneous NP_S_ synthesis through hydrogel networks. However, the use of reducing substance agent of NaBH_4_ is potentially causing cytotoxic process. For the in ex-situ method, previously synthesized AgNPs were combined with the ingredient polymeric materials before crosslinking operation of polymeric substances. On cross-linking formation, however, the assembly of NPs possibly occurs that it can obviously reduce the biological activity of AgNPs. Thus, the steadiness of suspension of NPs is a dire important parameter, the crust of AgNPs was modified by collagen, serum albumin prior formation of gel ^32^. Not long time ago, the development of human pathogens has rose globally producing a reduction in the efficiency of treatments for pathogenic infectivity ^33^. Candida albicans (*C. albican*) is widely dominating infected of fungal infection in human being. Without suitable treatment, life-injuring triggered by *C. albican* infection can occur, with a mortality of up to 50% ^34^.

The main obejetcive of recent work is to synthesize of silver nanoparticles–Loaded polyacrylamide/chitosan hydrogel nanocomposites to be utilized as antimicrobial and anti-cancer modulator. These hydrogel nanocomposites will be synthesized by gamma irradiation at large range of gamma irradiation. In addtion, various techniques will be utilized to charaterize these hydrogel nanoconposites such as FTIR, TGA, EDX, SEM and TEM. Further, antioxidant of activity of hydrogel and hydrogel nanocomposites will be studied. These hydrogel nanocomposites will be examained as an antifungal against *C. albicans* and anticancer modular as well.

## Materials and experimental part

### Materials

AgNO_3_ powder (purity of > 99.9%) and chitosan (M_w_ = 100–300 kDa and 75–85% deacetylated) supplied from Sigma-Aldrich Co. (USA). acrylamide monomer (purity of > 99%) and 0.5 McFarland standards were purchased from Sigma-Aldrich Co. (Germany)._._ Glacial acetic acid (purity of > 96%) was purchased from El-Nasr Pharmaceutical Chemicals Co. (Egypt). MTT (3-(4, 5-dimethylthiazol-2yl)-2, 5-diphenyltetrazolium bromide) supplied from Abcam company (USA) and (isopropano/HCl) (l/0.1 N) source is spectrum chemical MFG.corp (USA).

### Preparation of hydrogel nanocomposites

**Fig. 1:**
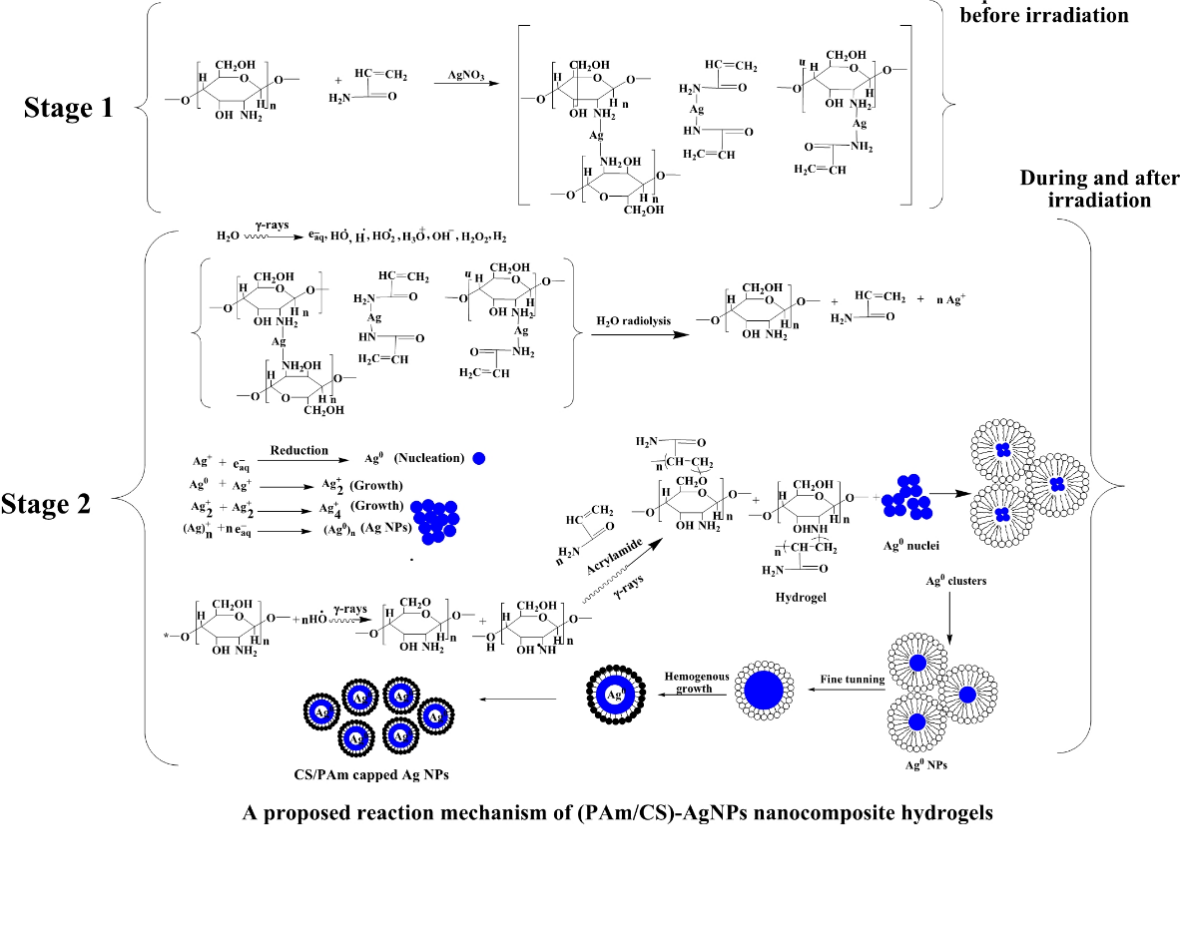
A suggested reaction mechanism of (PAm/CS)-AgNPs hydrogel nanocomposites.

A 1–3 g of chitosan (CS) powder was irradiated to 5 KGy after that dissolved in 100 ml of distelled water with 1% glacial acetic acid. 30 g of acrylamide (Am) monomer is dissolved in 97–99 mL of distilled water. The mixture of (Am/CS) is stirred well by magnetic stirrer of speed 120 rpm at 70 °C for 24 h to guarantee the complete dissolution of binary mixture. 0.1 g of AgNO_3_ is added to binary mixture of (Am/CS)and stirred for 24 at room temperature in dark container. The solutions are irradiated to a wide range of radiation dose from 5 to 15 kGy with dose rate 1 kGy/h and is ^60^Co as the main source of gamma rays (Indian cell, Egypt). The details of preparation conditions are listed in Table 1. The purpose of exposure to gamma irradiation is conversion of AgNO_3_ to Ag nanoaprticles (AgNPs) and to form a networks of (PAm/CS). A proposed reaction mechanism of AgNPs formation into hydrogel nanocomposites is shown in Fig. 1. The stage 1 illustates the intermediate state of complex formation with Am and CS before exposing to gamma irradiation intermediate complex ^35,36^. In addition, the stage 2 refers to the reaction between the consituent of Am and consituent of CS during exposing to gamma irradiation is graft copolymerization, and reduction of Ag^+^ cations and formation of stable AgNPs^37,38^.

**Table 1:** Preparation conditions of (PAm/CS)-AgNPs hydrogel nanocomposites.

Sample code	Synthesis conditions			Dose (kGy)
	PAm (g/ml)	CS (g/ml)	Ag (g/ml)	
(PAm/CS)-(0.3/0.01)-5	0.3	0.01	0.001	5
(PAm/CS)-(0.3/0.01)-10	0.3	0.01	0.001	10
(PAm/CS)-(0.3/0.01)-15	0.3	0.01	0.001	15
(PAm/CS)-(0.3/0.02)-5	0.3	0.02	0.001	5
(PAm/CS)-(0.3/0.02)-10	0.3	0.02	0.001	10
(PAm/CS)-(0.3/0.02)-15	0.3	0.02	0.001	15
(PAm/CS)-(0.3/0.03)-5	0.3	0.03	0.001	5
(PAm/CS)-(0.3/0.03)-10	0.3	0.03	0.001	10
(PAm/CS)-(0.3/0.03)-15	0.3	0.03	0.001	15

### Swelling (%) and gelation (%) estimation

The swelling (%) of hydrogel and hyhdrogel nanocomposites were estimated by dipping the dried hydrogel nanocomposites in distilled water for a given time at room temperature, and followed by weighing step. The value of swelling (%) was estimated according to Eq. (1)

1 $$Swelling \left(\%\right)= \frac{{W}_{t}-{W}_{0 }}{{W}_{0}} \times 100,$$

where *W*_*t*_ refers to weight swollen hydrogel or hydrogel nanocomposites, *W*_*o*_ is the weight of dry hydrogel before swelling step. Extraction of sol fraction was carried out by Soxhlet apparatus for 3 h in distilled water at 50 °C. the hydrogel and hydrogel nanocomposites were dried at 50 °C in oven for three days until reaching a constant weight. The value of gelation (%) was calculated gravimetrically by utilizing Eq. (2)

2 $$Gelation \left(\%\right)=\frac{{W}_{d}}{{W}_{0}} \times 100$$

where *W*_*d*_ refers to the weight of dry hydrogel and hydrogel nanocomposites after extraction step; and *W*_*o*_ is the weight of the dry hydrogel before extraction step.

### Characterization of prepared hydrogel and hydrogel nanocomposites

The infrared spectra were examined by Fourier transform infrared (FTIR) spectrophotometer, Perkin Elmer, USA, from 4000 to 400 cm^−1^. The morphology of samples were carried out by scanning electron microscope (SEM), Model ZEISS-EVO15-UK. The thermogravimetric analysis device (TGA) is utilized to investiagte the thermal breakdown of samples using a Shimadzu (Japan) TG-50 equipment. The heating was conducted in a N_2_ gas atmosphere at temperatures ranging from ambient temperature to 800 °C at a heating rate of 10 °C/min^1^. To inhibit heat oxidation of samples, the N_2_ gas flow was kept constant at around 20 (mL/min).The studied samples’ X-ray diffraction (XRD) patterns were invesitigated utilizing an X-ray diffractometer (a Shimadzu XRD 600). On the diffractometer utilizing a CuK_α_ radiation source, a generator voltage of 40 kV, a generator current of 40 mA, and a wavelength of 0.1546 nm, XRD patterns were recorded at a scan rate of 5° min^1^. All diffraction patterns were determined at room temperature and under known conditions. The energy dispersive X-ray (EDX) microanalysis is an elemental analysis device, which declares the elements found in the specimens was conducted utilizing an EDX unit (Zeiss Smart EDX, UK).Transmission electron microscopy (TEM), model JEM 100CS, Joel electron microscopy, Japan are utilized to investigate formation of AgNPs. In addition, the TEM images were acrried out at 80 kV.

### Viable cell counts of *C. albicans* after treatment with hydrogel and hydrogel nanocomposites

The antifungal property of hydrogel nanocomposites on the viability of *C. Albicans* was evaluated^39^. The overnight *C. albicans* culture was additionally incubated for 2 h at 37 °C. The *C. albicans* count was regulated to 0.4 McFarland standards equal (1 × 10^4^ cells/mL) at 600 nm. 5.0 mL of nutrient agar broth was inoculated with *C. albicans*. A volume of 0.5 mL of the nanocomposite at various concentrations of 0.01, 0.02, and 0.03 (mg/mL) have been adjoined to inoculated broth vaila. The vails were incubated at 37 °C for 24 h. Tenfold serial diluted concentrations of solution of stock are adapted to get diluted solutions from 10^–1^ to 10^–5^. The volume of 100 µL from the earlier three diluted solutions are conducted on plates of nutriential agar and kept at 37 °C for 24 h. The evalulation is conducted in three times. Colonies are accurately numbered and enumerated in units of CFU/mL.

### Toxicity test (MTT test)

A responsive in vitro test (MTT test) was conducted to evaluate cell proliferation or apoptotic cell death. MTT (3-(4, 5-dimethylthiazol-2yl)-2, 5-diphenyltetrazolium bromide) conversion and detection are applied. As proved by Abd-Allah and Fathy, 2022 ^40^; Hela carcinoma cells were cultured for 48 h in a 5% CO_2_ incubator utilzing a 1:1 ratio of Ag-(PAm/CS) Hydrogel nanocomposites (50, 25, 12.5, 6.25, 3.125 µg/ml). To start the colouring process, 0.5 mg MTT/mL was empolyed. Each cell was given 0.5 (mg/mL) MTT and grown for 48 h in a 5% CO_2_ gas incubator. The cells were then centrifuged, and the medium was removed by spinning (1500 rpm) for 5 min. Each control and sample tube received (500/l) of (isopropanol/HCl) mixture, which was then stirred to solubilize the formazan crystals and pelleted by spinning (1500 rpm for 5 min). The supernatants were collected, and the absorbance of light at a fixed wavelenghth λ = 560 nm was conducted. A blank containing only (isopropanol/HCl) was tested and the results were abstracted from all values. The percnetgae of alive cells were determined from Eq. (3).

3 $$\left(\text{Alive cells}\right)\%=\left(\frac{\text{test absorption}-\text{blank absorption}}{\text{control absorption}-\text{blank absorption}}\right)\times 100$$

### Antioxidant assay

DPPH radical scavenging activity of (PAm/CS) hydrogel and (PAm/CS)-Ag NPs hydrogel nanocomposite In brief, each 2.5-cm-diameter spherical specimen was subjected to a 10 ml methanol extraction, followed by one hour of agitation in a water bath. An aliquot of the material was then diluted with 10 ml of methanol. The reaction mixture, composed of sample (1.0 ml) and DPPH (3.0 ml) 3.0 ml of the DPPH with 1.0 ml of the sample, which had a concentration of 0.1 mM in methanol. The absorbance of the reaction mixture was meas-ured after a duration of 30 min, with precautions taken to protect it from exposure to light. The solution was allowed to undergo incubation for a duration of thirty minutes at ambient temperature under dark conditions. Subsequently, the 517 nm absorbance was read. The calculation was performed using the Eq. (4).

4 $$\left(\boldsymbol{\%}\boldsymbol{ }scavenging \right)\%=\left(\frac{{A}_{b}-{A}_{s}}{{A}_{b}}\right)\times 100$$

where *A*_*b*_ and *A*_*s*_ are the absorbance of mixture before and after to exposure to 30 min.

### Gene expression of PDGFR-β, Bcl2, Cathepsine, MMP-2 and P53 genes

Gene expression of PDGFR-β, Bcl2, Cathepsine, MMP-2 and P53 genes was conducted utilizing an Applied Biosystems 7500 to identify expression of PDGFR-β, Bcl2, Cathepsine, MMP-2 and P53 genes. Every response was completed triplicates. For 40 cycles, the riding conditions were 15 s at 95 degrees Celsius and one minute at 60 degrees Celsius. For relative measurement of gene expression, the Ct appraoch was utilized. To standardise the relative abundance of the PDGFR and P53 genes^38^, Taqman mRNA assays for glyceraldehyde-3-phosphate dehydrogenase protein were used ^41^.

### ELISA detection of NF-κB, PI3K, AKT and mTOR levels

HeLa cells were plated in 96-well plates at a density of 1 × 10^5^ cells per well, treated with imperatorin at 12.57 µg/ml of (Pam/CS) AgNPs hydrogel nanocomposites for 48 h. The levels of NF-κB, PI3K, AKT and mTOR in the culture supernatant were evaluated utilizing an ELISA kit (St John’s Laboratory Ltd., Knowledge Dock Business Centre, London, UK) and the manufacturer’s instructions.

### Statistical assessment

The data was statistically analyzed utilizing one-way ANOVA (P < 0.05) and Duncan’s test ^42,43^. SPSS version 20 was exploited to determine and analyze the results of the investigation.

## Results and discussion

**Fig. 2:**
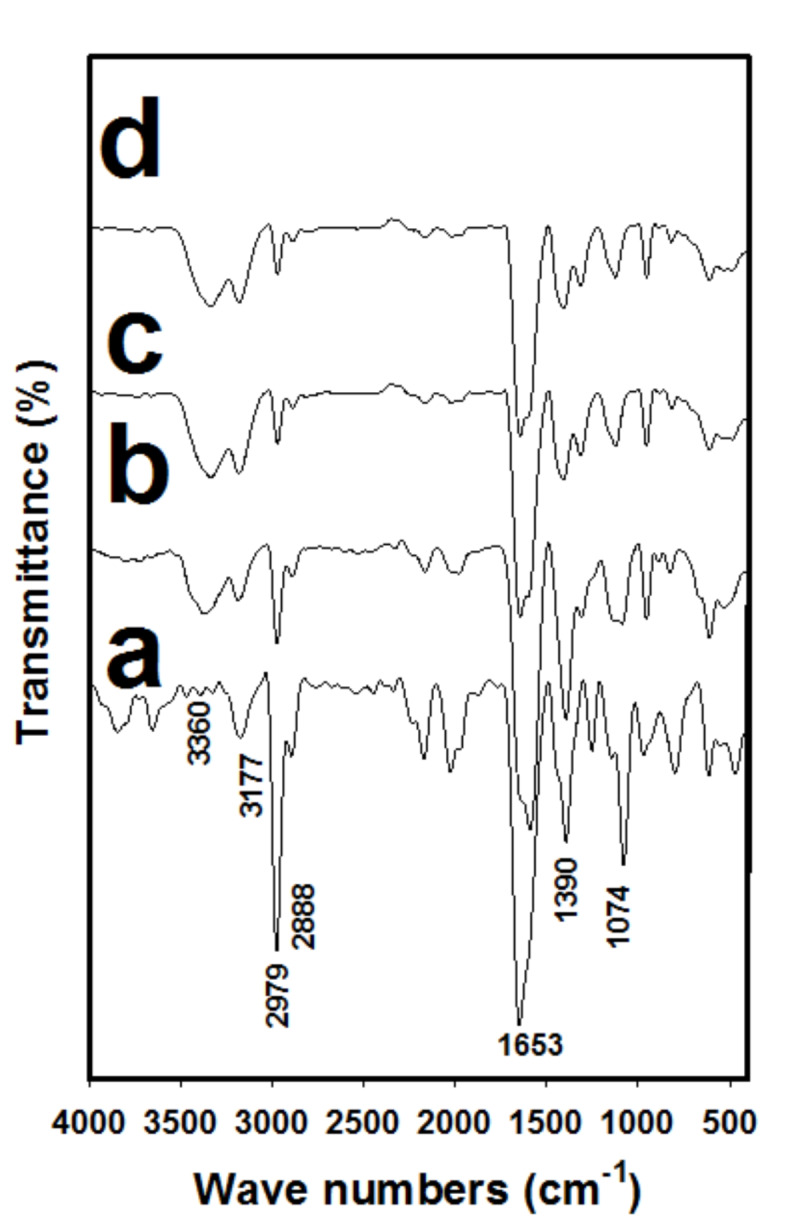
FT-IR spectra analysis of (**a**) (PAm/CS) hydrogel, (**b**) (PAm/CS)-(0.3/0.01)-5, (**c**) (PAm/CS)-(0.3/0.01)-10 and (**d**) (PAm/CS)-(0.3/0.01)-15 AgNPs hydrogel nanocomposites. Note, (PAm/CS) hydrogel was added for spectra clarification.

Figure 2a–d exposes FT-IR analyses of (PAm/CS) hydrogel, (PAm/CS)-(0.3/0.01)-5, (PAm/CS)-(0.3/0.01)-10 and (PAm/CS)-(0.3/0.01)-15AgNPs hydrogel nanocomposites, respectively. A peak at 1074 cm^−1^ for C–O-C stretching, which is characteristic peak for existnce of CS^44,45^. In addition, a stretching vibration for OH groups shows at 3360 cm^−1^^46^. A peak at 1390 cm^−1^ is referring to CH rocking of the CS ring. Peaks appear at 2979 and 2888 cm^−1^ are for symmetric and asymmetric groups of C–H. A characteristic peak appears at 1653 cm^−1^ for C=O group of PAm. It is noticed that, a peak appears at 3177 cm^−1^ for PAm is attributed to NH_2_^45^. It is seen from Fig. 2b–d that peaks appear at 2979 and 2888 cm^−1^ of symmetric and asymmetric CH reduces after adding Ag^+^ ions to in-situ solution to form hydrogel nanocomposites. Furthermore, a intensity of C=O peak reduces by augmenting the quantity of Ag^+^ cations into the hydrogel preapred. This confirms the existance of Ag^+^ cations into hydrogel structures. Moreover, it can be observed the peak intensity appears at 3360 cm^−1^ (OH) augments and an intensity of peak (NH_2_) reduces. This may be assigned to graft copolymerization of PAm occurred on NH_2_ more than on OH of CS is attributed to addition of Ag^+^ cations, while graft copolymerization of PAm before adding Ag^+^ cations may occur on both sites of NH_2_ and OH for CS as proposed reaction mechanism of Fig. 1. This a proposed reaction mechanism copes up with reported works in literature elsewhere ^45,46^. Moreover, it can be deduced that the existince of Ag into reaction media of sol may prevent the graft copolymerization process onto OH site of CS.

**Fig. 3:**
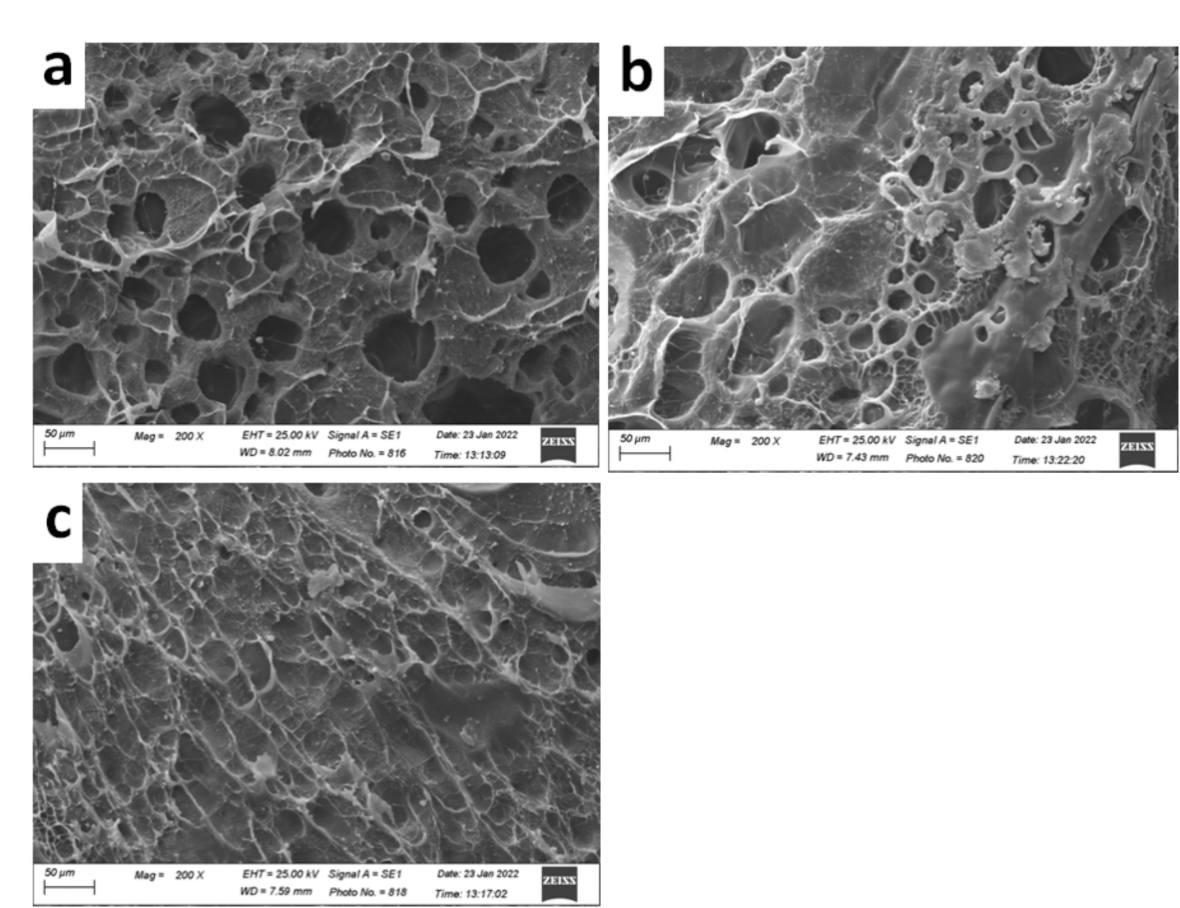
SEM photomicrographs of three AgNPs hydrogel nanocomposites (**a**) (PAm/CS)-(0.3/0.01)-10, (**b**) (PAm/CS)-(0.3/0.02)-10 and (**c**) (PAm/CS)-(0.3/0.03)-10.

Figure 3a–c shows SEM photomicrographs of three AgNPs hydrogel nanocomposites; (PAm/CS)-(0.3/0.01)-10, (PAm/CS)-(0.3/0.02)-10 and (PAm/CS)-(0.3/0.03)-10, accordingly. Generally, it is observed that the morphology of three AgNPs AgNPs hydrogel nanocomposites are unique in their general properties of morphology. It can be noticed the pores depth and their distribution which are approximately homogeneous as exposed in Fig. 3a. These pores diminish gradually by augmenting the content of CS into AgNPs hydrogel nanocomposites formed as appearing obviously in Fig. 3c. In addition, the morphology of (PAm/CS)-(0.3/0.02)-10 AgNPs hydrogel nanocomposites in Fig. 3b shows the critical composition among three hydrogel nanocomposites.

**Fig. 4:**
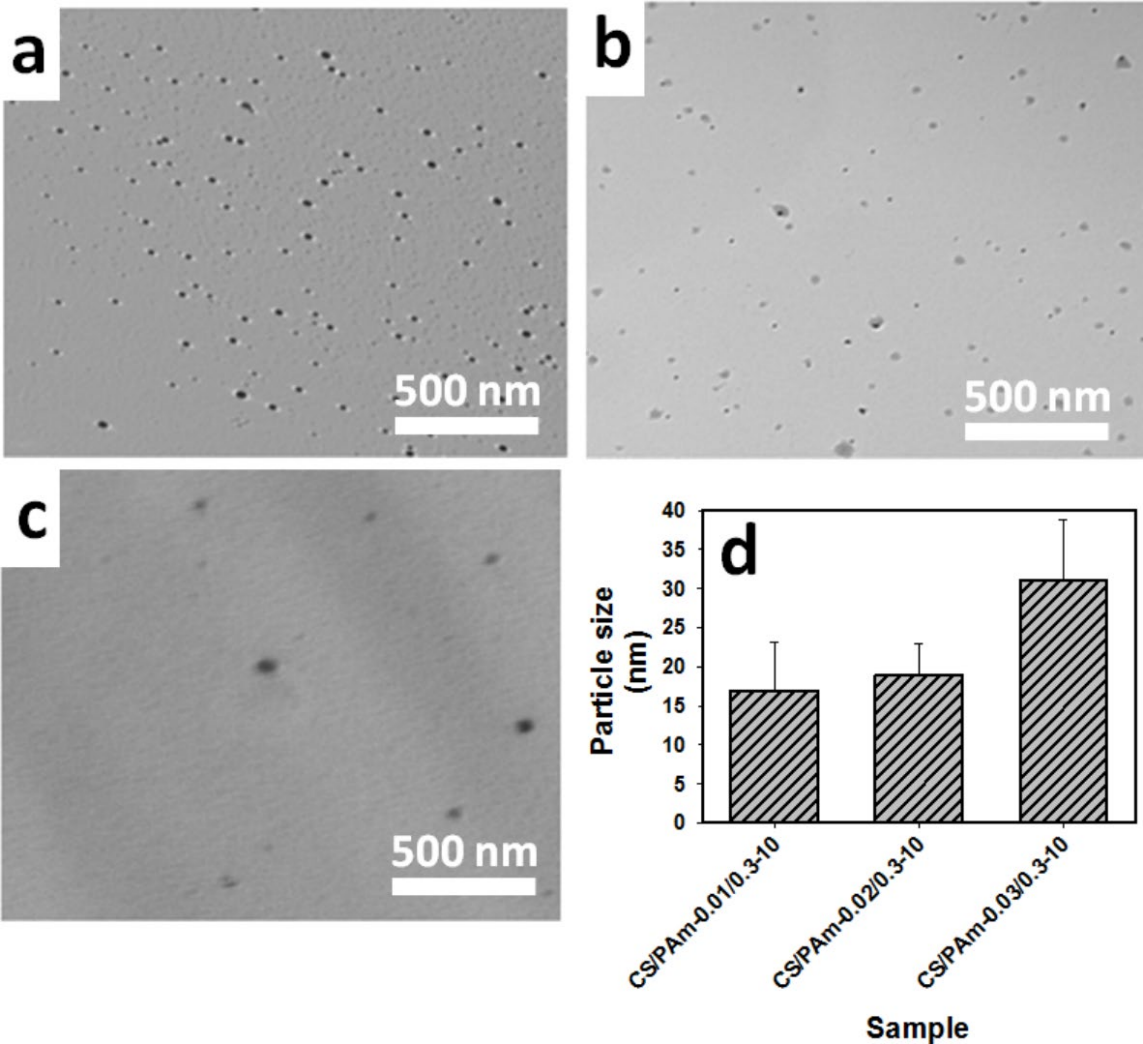
TEM photomicrographs of three AgNPs hydrogel nanocomposites (**a**) (PAm/CS)-(0.3/0.01)-10, (**b**) (PAm/CS)-(0.02/0.3)-10, (**c**) (PAm/CS)-(0.3/0.03)-10 and (**d**) effect of CS concentration on particle size (particle sizes were estimated from TEM images as average values followed by stadandbader devaition.

Figure 4a–d illustrates TEM photomicrographs of three AgNPs hydrogel nanocomposites (**a**) (PAm/CS)-(0.3/0.01)-10, (**b**) (PAm/CS)-(0.3/0.02)-10, (**c**) (PAm/CS)-(0.3/0.03)-10 and (**d**) effect of CS concentration, accordingly. Generally, it is seen that NPs formation of Ag distibuted in whole surface of (PAm/CS)-(0.3/0.01)-10 (Fig. 4a), (PAm/CS)-(0.3/0.02)-10 (Fig. 4b) and (Fig. 4c) (PAm/C)-(0.3/0.03)-10 AgNPs hydrogel nanocomposites. Moreover, it was observed from Fig. 4d that the particle size of Ag increases by increasing the concentration of CS into materix of hydrogel nanocomposites. In addition, the average particle size are 16.4 ± 6, 19 ± 4 and 31 ± 7 nm for (PAm/CS)-(0.3/0.01)-10, (PAm/CS)-(0.3/0.02)-10 and (PAm/CS)-(0.03/0.3)-10, accordingly. Moreover, the elemental analysis for three hydrogel nanocomposites was listed in Table 2. Overall, it wasobserved that the exsitence of Ag into structure of all hydrogel nanocomposites. The percentage of Ag in three AgNPs hydrogel nanocomposites is approximately a fixed at a given value as aforementioned in preapartion conditions of experimenatal section.

**Table 2:** Formulas realtionships among swelling (%), gelation (%), composition and radiation dose; and elemental analysis by EDX for AgNPs hydrogel nanocomposites.

Sample	Formulas of swelling versus dose			
(PAm/CS)-(0.3/0.01)	*S* = 1.8*D*^2^ − 48.6*D* + 517 ***R***^***2***^ = 0.99			
(PAm/CS)-(0.3/0.02)	*S* = 1.56 *D*^*2*^ − 44.6*D* + 486 ***R***^***2***^ = 0.99			
(PAm/CS)-(0.3/0.03)	*S* = 0.78*D*^*2*^ − 30.3*D* + 494 ***R***^***2***^ = 0.99			

Sample	Formulas of swelling versus composition			
(PAm/CS)-(0.3/0.01)-5	*S* = − 855*C* + 351 ***R***^***2***^ = 0.95			
(PAm/CS)-(0.3/0.02)-5				
(PAm/CS)-(0.3/0.03)-5				
(PAm/CS)-(0.3/0.01)-10	*S* = − 630 *C* + 234 ***R***^***2***^ = 0.97			
(PAm/CS)-(0.3/0.02)-10				
(PAm/CS)-(0.3/0.03)-10				
(PAm/CS)-(0.3/0.01)-15	*S* = − 1170 *C* + 236 ***R***^***2***^ = 0.96			
(PAm/CS)-(0.3/0.02)-15				
(PAm/CS)-(0.3/0.03)-15				

Sample	Formulas of gelation (%) versus dose			
(PAm/CS)-(0.3/0.01)	*S* = 0.14 *D* + 13.8 ***R***^***2***^ = 0.94			
(PAm/CS)-(0.3/0.02)	*S* = 0.083 *D* + 19.12 ***R***^***2***^ = 0.97			
(PAm/CS)-(0.3/0.03)	*S* = 0.127 *D* + 20.47 ***R***^***2***^ = 0.97			

Sample	Formulas of gelation (%) versus composition			
(PAm/CS)-(0.3/0.01)-5	*G* = − 1452 *C*^*2*^ + 292 C + 6.41 ***R***^***2***^ = 0.99			
(PAm/CS)-(0.3/0.02)-5				
(PAm/CS)-(0.3/0.03)-5				
(PAm/CS)-(0.3/0.01)-10	*G* = − 1552 *C*^*2*^ + 306 C + 6.52 ***R***^***2***^ = 0.99			
(PAm/CS)-(0.3/0.02)-10				
(PAm/CS)-(0.3/0.03)-10				
(PAm/CS)-(0.3/0.01)-15	*G* = − 999 *C*^*2*^ + 229.8 *C* + 9.45 ***R***^***2***^ = 0.99			
(PAm/CS)-(0.3/0.02)-15				
(PAm/CS)-(0.3/0.03)-15				

Sample	Element (wt.%)			
	C	N	O	Ag
(PAm/CS)-(0.3/0.01)	34.41	19.91	45.14	0.54
(PAm/CS)-(0.3/0.02)	46.17	19.33	34	0.5
(PAm/CS)-(0.3/0.03)	47.18	22.13	30.18	0.51

Abbreviations of C, D, G and S refer to the composition, dose, gelation (%) and swelling (%), respectively.

**Fig. 5:**
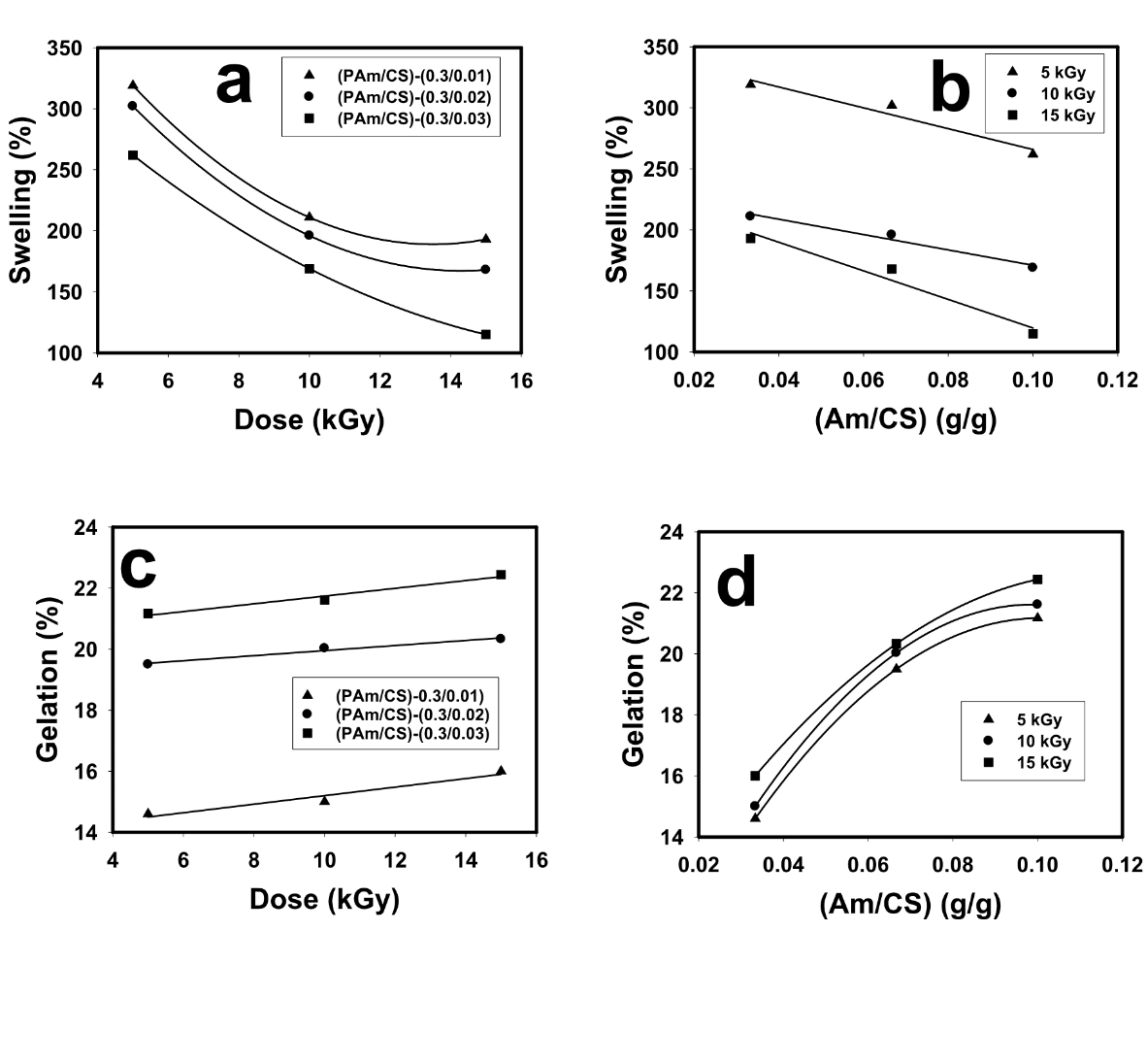
Influence of radiation dose and feeding composition of (Am/CS) on swelling (%) and gelation (%) of (PAm/CS) hydrogel and (PAm/CS)-AgNPs hydrogel nanocomposites.

Figure 5a–d shows the influence of radiation dose and feeding composition of (Am/CS) on swelling (%) and gelation (%) of hydrogel and AgNPs hydrogel nanocomposites. It is observed from Fig. 5a that the swelling (%) decreased by increasing the radiation dose. This is attributed to increasing the crosslinking density of networks as results of increasing the radiation dose, consequently making a decrease in amount of water molecules absorption among networks^43^. The line regression parameters of experimental results for the relationship between the swelling (%) and radiation dose are listed in Table 2. It was observed values of R^2^ for all regressions are good. Thus, the relationship between the swelling (%) and radiation is very strong. Further, the results of Fig. 5a listed in Table 2 that showing the relationship between the swelling (%) and the radiation dose belongs to second linear regression. It was seen from Fig. 5b that the swelling (%) reduces by augmenting the feeding composition of (Am/CS) and relationship between the swelling (%) and feeding composition is first linear regression and the values of R^2^ indicate to the very strong relationship between them.

**Fig. 6:**
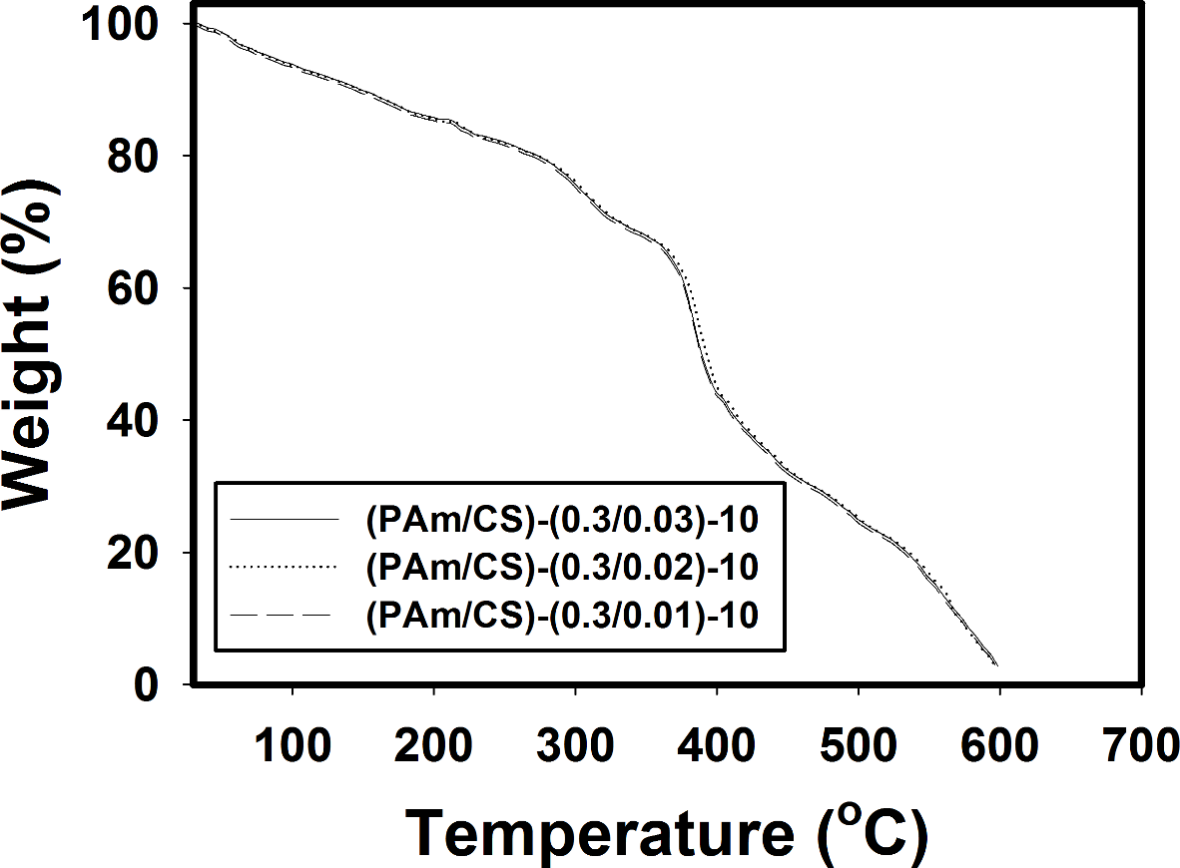
TGA thermograms of (**a**) (PAm/CS)-(0.3/0.01)-10, (**b**) (PAm/CS)-(0.3/0.02)-10, and (**c**) (PAm/CS)-(0.3/0.03)-10 AgNPs hydrogel nanocomposites.

Figure 6 shows TGA thermograms of (PAm/CS)-(0.3/0.01)-10, (PAm/CS)-(0.3/0.02)-10, and (PAm/CS)-(0.3/0.03)-10 AgNPs hydrogel nanocomposites**,** respectively**.** Generally, it was observed the weight (%) decreases by increasing the temperature. Overall, it is seen that the grafted copolymer synthesized utilizing diverse composition of CS relative to weight of PAm starts to loss the absorbed water molecules at 185 °C. The fundemental decomposition stage of the natural region into formed hydrogel occurs in range of temperature from 200 to 220 °C with a weight loss of ~ 71% that is referring to unsystematic chain breaking and CS deacetylation and decomposition of amide groups from grafted PAm ^44^. In third stage, a loss of weight around 8% occurs in the range of 373–435 °C that is attributed to the decomposition of the main backbone of grafted PAm onto CS. In conclusion, it can be deduced that the change in thermal decomposition is nonsignificant ^46^.

**Fig. 7:**
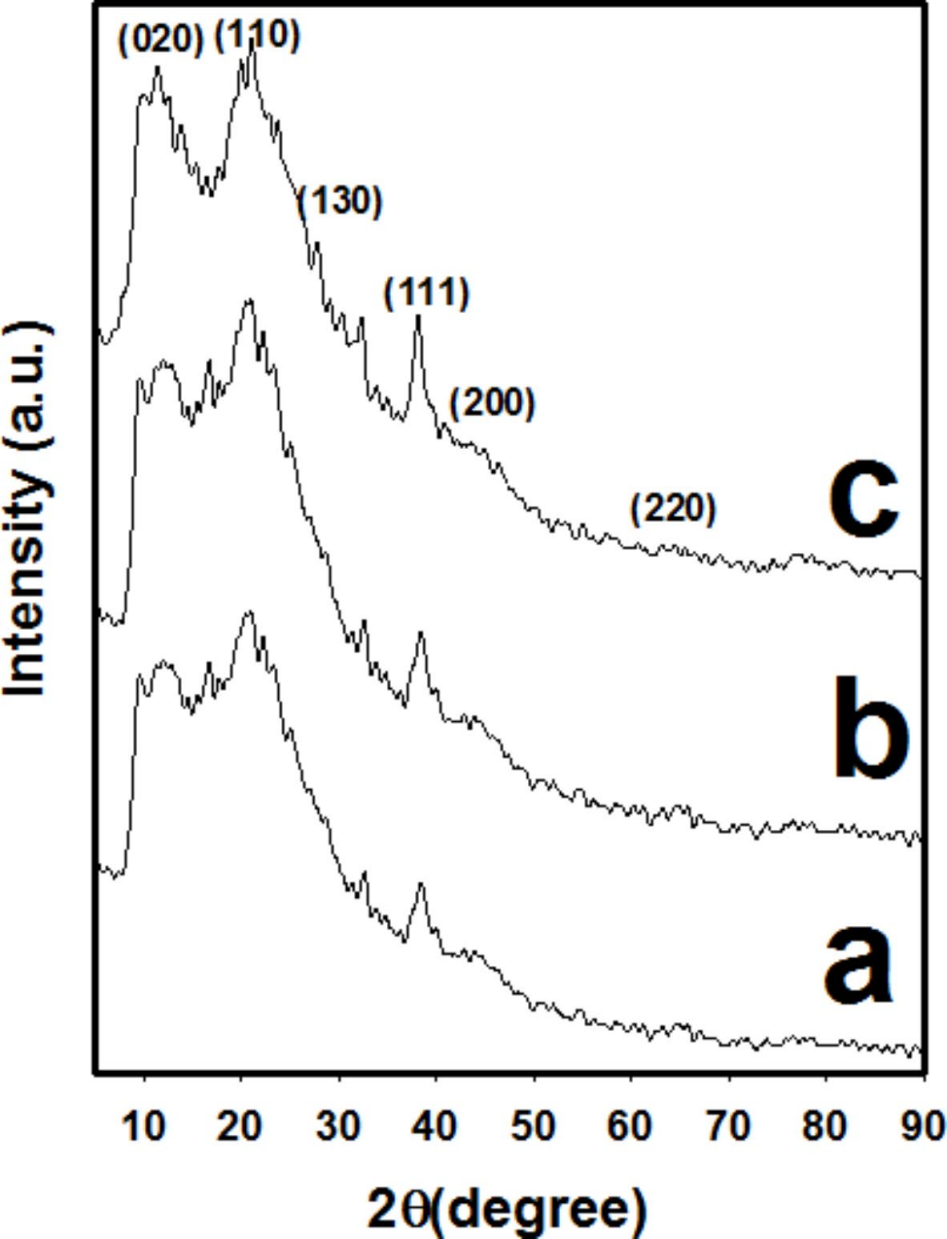
XRD patterns of (**a**) (PAm/CS)-(0.3/0.01)-10, (**b**) (PAm/CS)-(0.3/0.02)-10, and (**c**) (PAm/CS)-(0.3/0.03)-10 AgNPs hydrogel nanocomposites.

Figure 7 shows the XRD patterns of (a) (PAm/CS)-(0.3/0.01)-10, (b) (PAm/CS)-(0.3/0.02)-10, and (c) (PAm/CS)-(0.3/0.03)-10 AgNPs hydrogel nanocomposites, respectively. Either CS or PAm exposed the diffraction peaks at 2θ values of ~ 11.9°, ~ 19.7° and ~ 29.1° that can be assigned to (020), (110) and (130), accordingly^47^. The diffraction peaks at 11.7° (020) and 19.8° (110) represent the amorphous and the crystalline regions of PAm and CS^48,49^. Further, these values are good agreement with mentioned results in elsewhere^50,51^. Generally, the broadening of the peaks is attraibuted to the amorphous structure of the PAm and CS that overlapped typically^52^. Moreover, the diffraction pattern displays the characteristic peaks are at 2θ of ~ 38.8°, ~ 44.3° and ~ 63.6°, which are attributed to (111), (200) and (220) reflections of the face centered cubic (fcc) structure of metallic silver, accordingly^53^. XRD patterns of PAm/CS/AgNPs-composite hydrogel nanocomposites obvisudly refer to the formation of silver in a single phase inside the hydrogel nanocomposites. The outcome values are quitely agreement with that of the JCPDS card no. 89-3722^54^.

**Fig. 8:**
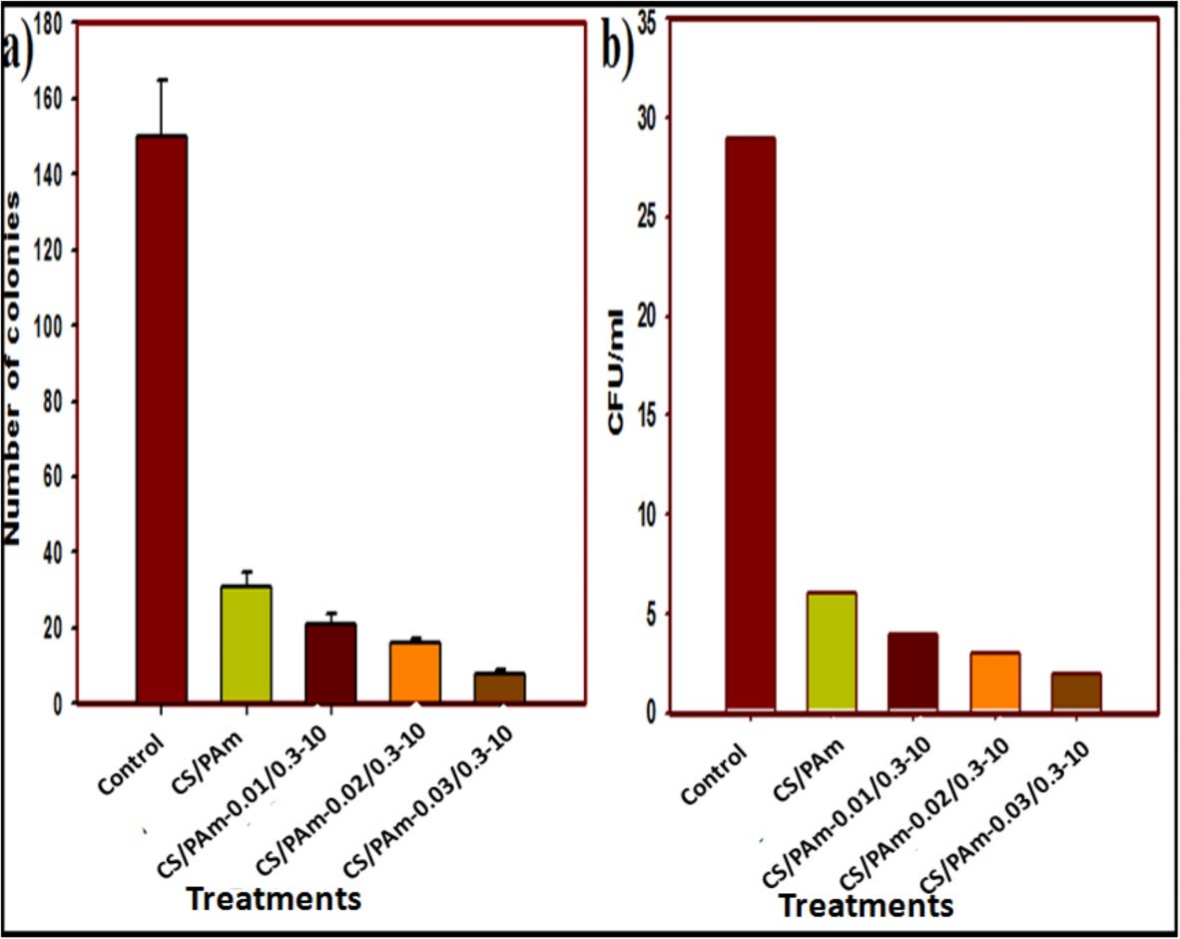
Influence of control, (PAm/CS) hydrogel, (PAm/CS)-(0.3/0.01)-10, (PAm/CS)-(0.3/0.02)-10 and (PAm/CS)-(0.3/0.03)-10 (**a**) AgNPs hydrogel nanocomposites on numbers of colons and (**b**) CFU/mL of *C. Albican*.

The number of live *C.* albicans cells per milliliter of culture was decreased, as estimated by CFU/mL, after overnight exposing to control, (PAm/CS) hydrogel, (PAm/CS)-(0.3/0.01) 10, (PAm/CS)-(0.3/0.02)-10, and (PAm/CS)-(0.3/0.03)-10 AgNPs hydrogel nanocomposites as shown in Fig. 8a,b, resepctively. It was observed from Fig. 8a that the results showed that the treatment was more effective at inhibiting the growth of *C.* albicans than the control sample. Figure 8b exposed that the antifungal property of the treatment was determined from 10^–4^ to 10^–6^ serial diluted concentations of *C. albicans* inoculum. In addition, all concentrations of the control, (PAm/CS) hydrogel, (PAm/CS)-(0.3/0.01)-10, (PAm/CS)-(0.3/0.02)-10, and (PAm/CS)-(0.3/0.03)-10 AgNPs hydrogel nanocomposites treatments decreased the number of colony-forming units of *C. albicans*. The CFU/mL of *C. albicans* with the treatment without CS registed the least colony number to be 6.2 × 10^5^. On the other hand, from Fig. 8b the CFU/mL decreased gradually with the augmenting in the concentration of CS recording the colony count of 4.2 × 10^5^, 3.2 × 10^5^, and 1.6 × 10^5^ CFU/mL, respectively. The *C. albicans* of control sample growth (non- treatment) shows a huge units that form colony (30 × 10^5^ CFU/mL). The influence of (PAm/CS)-AgNPs hydrogel nanocomposite concentration on HeLa cell viability was listed in Table 3. Moreover, It was noticed from Table 3 that the influence of (PAm/CS)-AgNPs hydrogel nanocompsoite on p53, Bcl2 and Cathepsine by utilizing MTT assay, it was observed that the (PAm/CS)-AgNPs hydrogel nanocomposite IC_50_ against cervical HeLa cancer cell line is 12.57 (µg/ml).

**Table 3:** The influence of (PAm/CS)-AgNPs hydrogel nanocomposite concentration on HeLa cell viability.

Conc. (µg/ml)	Abs1	Abs2	Abs3	Mean	SD	Viability (%)	IC50% (µg/ml)
0	0.31	0.32	0.33	0.32	0.007	100	12.57
6.25	0.29	0.30	0.30	0.30	0.006	91.81	
12.5	0.25	0.21	0.18	0.22	0.035	66.94	
25	0.11	0.13	0.14	0.13	0.014	39.38	
50	0.10	0.11	0.11	0.11	0.005	32.85	
100	0.08	0.08	0.09	0.08	0.004	25.39	

**Fig. 9:**
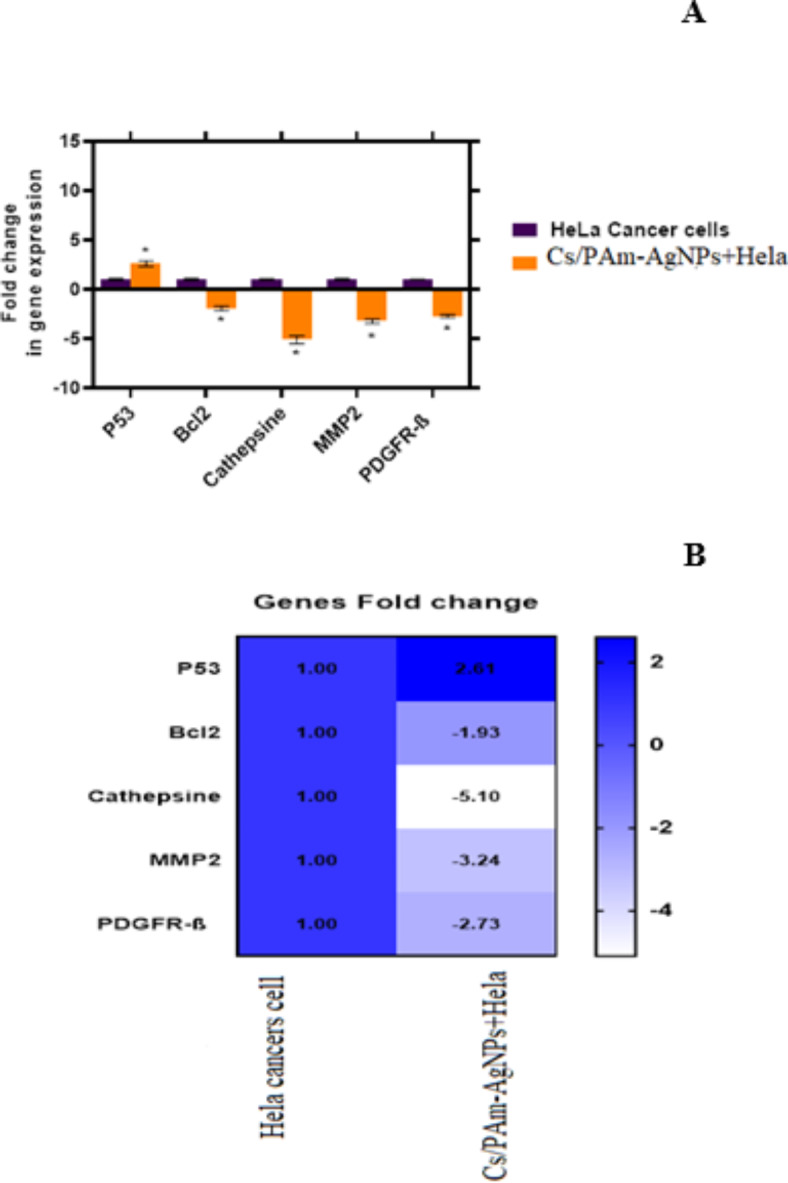
PDGFR-β, Bcl2, Cathepsine, MMP-2 and P53 fold change in HeLa malignant cell line post 48 h incubation with (PAm/CS)-AgNPs hydrogel nanocomposites. (**a**) Histogram and (**b**) Heatmap.

Figure 9a,b displayed that the anticancer activity of (PAm/CS)-AgNPs hydrogel nanocomposites against the HeLa cervical cancer cell line was studied utilzing the MTT assay and heatmap, accordingly. It was noticed from Fig. 9a that the MTT assay was utilized to determine the viability of HeLa cervical cancer cells treated with different amount of silver nanoparticle-loaded (PAm/CS) hydrogel nanocomposite. HeLa cells cultured in RPMI complete media were utilized as a positive control (100% viability). The concentration of (PAm/CS)-AgNPs hydrogel nanocomposites that prohbited the growth of cancer cells by 74.6% after 48 h of incubation was 100 µg/ml. In addition, lower concentrations of 50, 25, 12.5, and 6.25 µg/ml prohbited cell growth by 67.15%, 60.6%, 33.1%, and 8.2%, respectively. Furthermore, (PAm/CS)-AgNPs hydrogel nanocomposite exposed that an IC_50_ of 12.57 µg/ml against the HeLa cervical cancer cell line. Figure 9b illustrates that the influence of (PAm/CS)-AgNPs hydrogel nanocomposites on the expression of p53, Bcl2, cathepsin, MMP-2, and PDGFR-β was studied. Through the results, it was observed that (PAm/CS)-AgNPs hydrogel nanocomposites significantly decreased the expression of the genes Bcl2, cathepsin, MMP-2, and PDGFR-β in the HeLa cervical cancer cell line. Moreover, compared to the control group, the expression of Bcl2, cathepsin, MMP-2, and PDGFR-β was downregulated by 1.93-, 5.1-, 3.24-, and 2.73 fold, respectively. Besides, the expression of the p53 gene in HeLa cervical cancer cells augmented by 2.61fold after treatment with (PAm/CS)-AgNPs hydrogel nanocomposite.

**Fig. 10:**
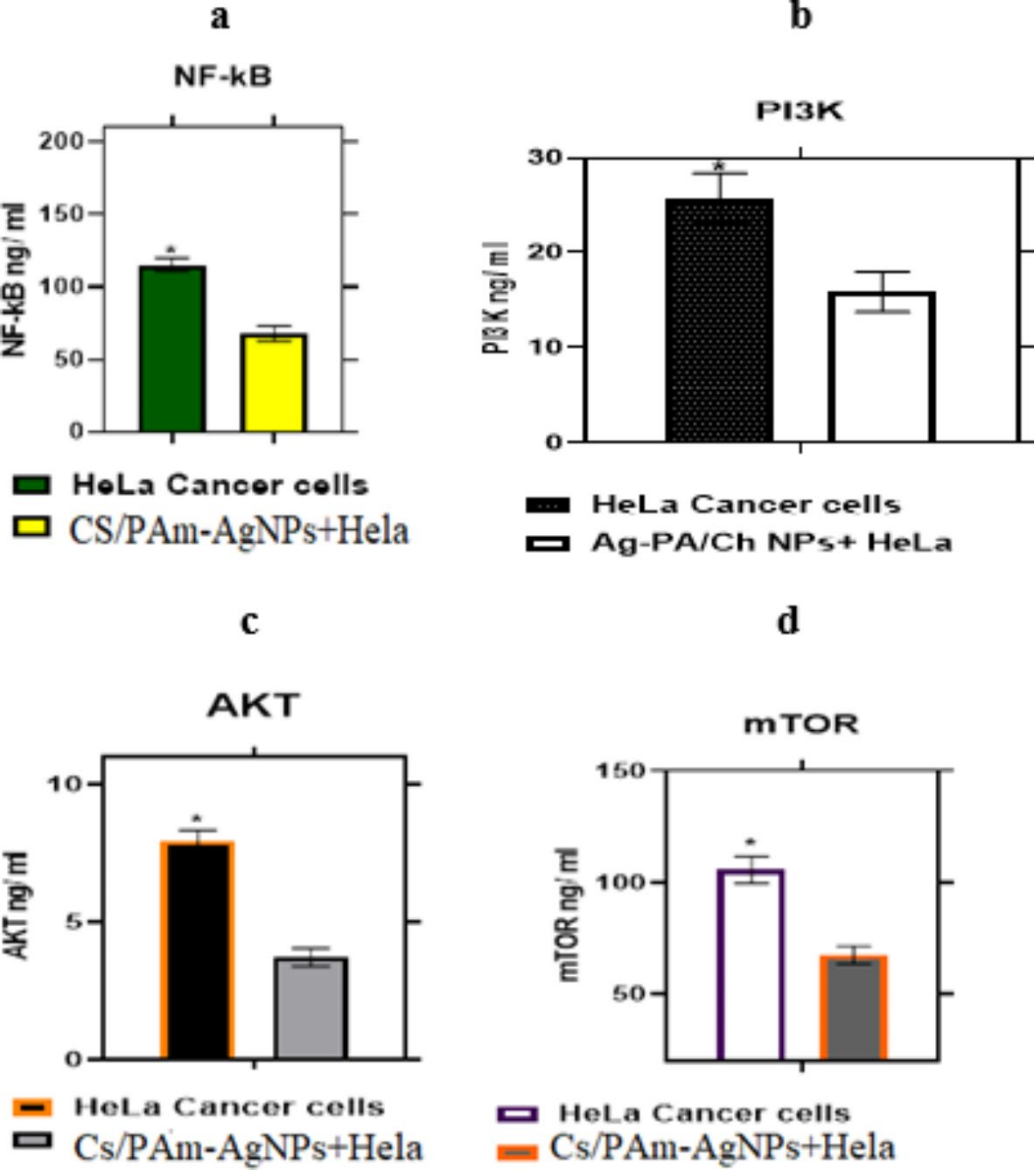
HeLa malignant cell line post 48h incubation with (PAm/CS)-AgNPs hydrogel nanocomposites levels of (**a**) NF-κB (**b**) PI3K (**c**) AKT and (**d**) mTOR.

Figure 10a–d displayed the influence of (PAm/CS)-AgNPs hydrogel nanocomposites on NF-kB, PI3K, AKT, and mTOR levels, accordingly. Generally, the findings declared that (PAm/CS)-AgNPs hydrogel nanocomposites significantly reduced the levels of NF-kB, PI3K, AKT, and mTOR in the cervical HeLa cancer cell linec compared to HeLa cancer cell line group, NF-kB, PI3K, AKT, and mTOR levels were reduced to 41.3%, 38.2%, 53.1%, and 36.1%, respectively. Consequntly, the (PAm/CS)-AgNPs hydrogel nanocomposites have signfcant influence on NF-kB, PI3K, AKT, and mTOR.

**Fig. 11:**
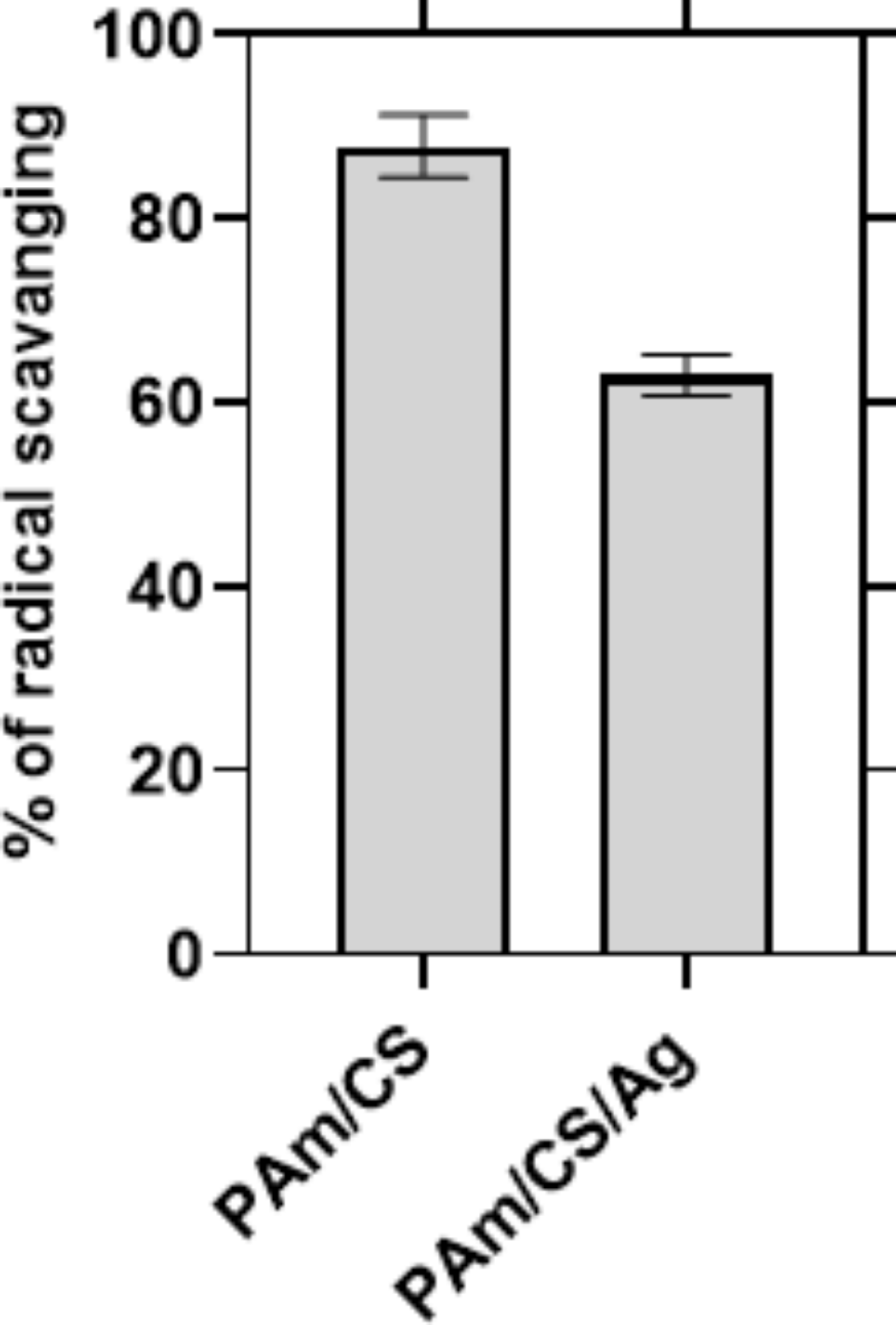
Antioxidant activity of (PAm/CS) and (PAm/CS)-AgNPs hydrogel nanocomposite.

Free radicals and reactive oxygen species are spontanously generated from physiological processes in the human body and their over-production beyond the capacity of the naturally available antioxidant defense system empoltyes to oxidative stress. Antioxidant compounds ohnbit oxidative deeroaration by scavenging free radicals, chelating catalytic metals and inhibiting lipid peroxidation^55^. They also represents as reducing agents with the ability to protonate oxidizing agents (e.g., hydroxyl radical) or to transfer electron or hydrogen atom to oxidants or free radicals ^56^. As recorded in this review, the assays based on DPPH radical, ABTS radical, oxygen radical, and ferric ion reducing antioxidant power were mentioned to be the most frequently utlized antioxidant methods ^57^. Figure 11 shows the antioxidant activity of (PAm/CS) and (PAm/CS)-AgNPs hydrogel nanocomposites in other words, the antioxidant activity means the % of radical scavenging of (PAm/CS) and (PAm/CS)-AgNPs hydrogel nanocomposite. Overall, it can be seen that the % of radical scavenging was 87.8% for (PAm/CS) hydrogel and 62.9% for (PAm/CS)-AgNPs hydrogel nanocomposite, indicating that the presence of Ag into the materix of hydrogel nanocomposite that reduces the antioxidant activity of the hydrogel.

## Conclusions

Hydrogel composites and nanocomposites were synthesized from Am, CS and sliver nitrate utilizing gamma irradiation from ^60^Co as the main source of gamma irradiation. Diverse parameters were used in the preaparation of silver nanoparticles-laoded hydrogel nanocomposites such influence of radiation dose and influence of CS concentration. Moreover, diverse techniques were utilized to characterize the hydrogel nanocomposites such FTIR, TGA, EDX, SEM and TEM. Through the results, it can be confirmed that the formation of silver nanoparticles-loaded hydrogel nanocompsoites. The antifungal activity of (PAm/CS)-(0.3/0.01)-10, (PAm/CS)-(0.3/0.02)-10 and (PAm/CS)-(0.3/0.03-10 AgNPs hydrogel nanocomposites on the viability of *C. albicans* was determined. The results declared the efficient microbial inhibition activity of treatment against *C. albicans* compared to the control. Further, the cytotoxicity of hydrogel nanocomposites against cervical cancer HeLa cell line was studied. The cytotoxicity of silver nanoparticles-loaded hydrogel nanocomposites on the prior cell line of cancer empolyed to inhibit growth of cell estimated by MTT test. The treatment of HeLa cancer cell with silver nanoparticles-loaded hydrogel nanocompsoites for 48 h confirmed a potential apoptotic activity by obvious upregulation of p53 gene expression. Moreover, the activity of anticancer was investigated by downregulation of platelet-based growth factor receptor beta (PDGFR-β), Bcl2, Cathepsine, and MMP-2 gene expression. The results of (PAm/CS)-AgNPs hydrogel nanocomposites showed that the anticancer, anti-metastatic and anti-angiogenesis activity, AgNPs influence was caused by downregulation of cathepsin D, which regulates protein synthesis, growth, cell proliferation, and survival. The antioxidant activity was investigated and the results showed antioxidant activity of (PAm/CS) hydrogel and (PAm/CS)-AgNPs hydrogel nanocomposite are 87.8% and 62.9%, respectively.

## Data Availability

The datasets used and/or analysed during the current study available from the corresponding author on reasonable request.

## References

[1] Harris, B. H. L., Macaulay, V. M., Harris, D.A., Klenerman, P., Karpe, F., Lord, S. R., Harris, A. L. & Buffa F. M. A Perfect storm for carcinogenesis. *Cancer. Metastasis. Rev. ***41,** 491–515 (2022). 10.1007/s10555-022-10046-2 PMC9470699 36038791

[2] Karidio, D. I. & Sanlier, S. H. Reviewing cancer’s biology: an eclectic approach. *J. Egypt. Natl. Cancer Inst. ***33**, 32 (2021). 10.1186/s43046-021-00088-y PMC13316913 34719756

[3] Kazeminava, F., Arsalani, N. & Akbari, A. POSS nanocrosslinked poly (ethyleneglycol) hydrogel as hybrid material support forsilver nanocatalyst. *Appl. Organometal. Chem. * e4359 (2018).

[4] Drakopoulou, E., Anagnou, N. P. & Pappa, K. I. Gene therapy for malignant and benign gynaecological disorders: A systematic review of an emerging success story. *Cancers ***14**, 3238 (2022). 35805007 10.3390/cancers14133238 PMC9265289

[5] Kazeminava, F. et al. Crosslinking chitosan with silver-sulfur doped graphene quantum dots: An Efficient antibacterial nanocomposite hydrogel films. *J. Polym. Environ. ***32**, 213–224 (2024).

[6] Haroun, A. A., Ahmed, E. F. & Abd El-Ghaffar, M. A. Preparation and antimicrobial activity of poly (vinyl chloride)/gelatin/montmorillonite biocomposite films. *J. Mater. Sci. Mater. Med*. **22**, 2545–2553 (2011). 10.1007/s10856-011-4437-x 21909641

[7] Haroun, A. A., Abdelghaffar, F. & Hakeim, O. A. Preparation of chitosan/hyperbranched polyester/cobalt composite for Acid Blue 277 dye adsorption. *Biointer. Res. Appl. Chem. ***11**, 11653–11665 (2021).

[8] Cui, I., Rong, J., Cong, C. & Ovalbumin, O. Loaded CuS nanoparticle for tumour modulation and enhanced immunotherapy of osteosarcoma. *Micro. Nano. Lett. ***17**, 101–106 (2022).

[9] Donati, G. & Amati, B. MYC and therapy resistance in cancer: risks and opportunities. *Mol. Oncol. ***16**, 3828–3854 (2022). 36214609 10.1002/1878-0261.13319 PMC9627787

[10] Tissot, S. et al. Transmissible cancer evolution: The under-Estimated role of environmental factors in the “perfect storm” theory. *Pathogens. ***11**, 241 (2022). 35215185 10.3390/pathogens11020241 PMC8876101

[11] Haroun, A. A., Abo-Zeid, M. A., Youssef, A. M. & Gamal-Eldeen, A. In vitro biological study of gelatin/PLG nanocomposite using MCF-7 breast cancer cells. *J. Biomed. Mater. Res. Part A ***101A**, 1388–1396 (2013). 10.1002/jbm.a.34441 23077120

[12] Wu, J., Chen, A., Qin, M., Huang, R., Zhang, G., Xue; B., Wei, J., Li, Y., Cao, Y. & Wang, W. Hierarchical construction of a mechanically stable peptide–graphene oxide hybrid hydrogel for drug delivery and pulsatile triggered release in vivo. *Nanoscale. ***7,**1655–1660 (2015). 10.1039/c4nr05798h 25559308

[13] Lilhare, S., Mathew S. B., Singh A. K. & Carabineiro S. A. C. Aloe vera functionalized magnetic nanoparticles entrapped Ca alginate beads as novel adsorbents for Cu(II) removal from aqueous solutions. *Nanomaterials (Basel). ***12**, 2947 (2022). 10.3390/nano12172947 PMC9457615 36079984

[14] Capanema, N. S. V. et al. Physicochemical properties and antimicrobial activity of biocompatible carboxymethylcellulose-silver nanoparticle hybrids for wound dressing and epidermal repair. *J. Appl. Polym. Sci. ***135**, 45812 (2018).

[15] Haroun, A. A., Mashaly, H., Helmy, H. & Kamel, M. Kinetic study of gelatin/chitosan based nanocomposites for acid red 150 dye adsorption using ultrasonic energy. *Egypt. J. Chem. ***60**, 41–54 (2017).

[16] Alarcon, E. I. et al. Safety and efficacy of composite collagen-silver nanoparticle hydrogels as tissue engineering scaffolds. *Nanoscale. ***7**, 18789–18798 (2015). 26507748 10.1039/c5nr03826j

[17] Haroun, A. A., Ahmed, E. F., El-Halawany, N. R. & Taie, H. A. A. Antimicrobial and antioxidant properties of novel synthesized nanocomposites based on polystyrene packaging material waste. *IRBM* **34**, 206–213 (2013).

[18] Lengert, E., Parakhonskiy, B., Khalenkow, D., Zecic, A., Vanghell, M., Moreno, J. M. M., Braeckman, B. P. & Skirtach, A. G. Laser-induced remote release in vivo in *C. elegans* from novel silver nanoparticles-alginate hydrogel shells. *Nanoscale. ***10**, 17249–17256 (2018). 10.1039/c8nr00893k 30191939

[19] Hou, C., Ma, K., Jiao, T., Xing, R., Li, K., Zhou, J. & Zhang, L. Preparation and dye removal capacities of porous silver nanoparticle-containing composite hydrogels via poly(acrylic acid) and silver ions. *RSC Adv. ***6**, 110799–110807 (2016).

[20] Zhang, X., Malhotra, S., Molina, M. & Haag, R. Micro- and nanogels with labile crosslinks—from synthesis to biomedical applications. *Chem. Soc. Rev.* **44**, 1948–1973 (2015). 25620415 10.1039/c4cs00341a

[21] Shah, S., Sasmal, P. K. & Lee, K.-B. Photo-triggerable hydrogel–nanoparticle hybrid scaffolds for remotely controlled drug delivery. *J. Mater. Chem. B. ***2**, 7685–7693 (2014). 25580246 10.1039/C4TB01436G PMC4285771

[22] Radosavljević, A., Spasojević, J., Krstić, J. & Kačarević-Popović, Z. Hydrogel nanocomposites obtained by gamma irradiation. In *Cellulose-Based Superabsorbent Hydrogels. Polymers and Polymeric Composites: A Reference Series* (eds Mondal, M.) (Springer, 2019).

[23] Campbell, S. B., Patenaude, M. & Hoare, T. Injectable superparamagnets: Highly elastic and degradable poly(N-isopropylacrylamide)–superparamagnetic iron oxide nanopart*icle (SPION) compo*site hydrogels. *Biomacromolecules.* **14**, 644–653 (2013). 23410094 10.1021/bm301703x

[24] Cheng, Z., Chai, R., Ma, P., Dai, Y., Kang, X., Lian, H., Hou, Z. & Li, C. Lin. Multiwalled carbon nanotubes and NaYF4:Yb^3+^/Er^3+^ nanoparticle-doped bilayer hydrogel for concurrent NIR-triggered drug release and up-conversion luminescence tagging. *Langmuir. ***29,** 9573–9580 (2013). 10.1021/la402036p 23829598

[25] Kuang, H. et al. Injectable and biodegradable supramolecular hydrogels formed by nucleobase-terminated poly(ethylene oxide)s and α-cyclodextrin. *J. Mater. Chem. B. ***2**, 659–667 (2014). 32261284 10.1039/c3tb21475c

[26] Antezana, P. E., Municoy, S., Pérez, C. J. & Desimone, M. F. Collagen hydrogels loaded with silver nanoparticles and cannabis sativa oil. *Antibiotics. ***10**, 1420 (2021). 34827358 10.3390/antibiotics10111420 PMC8615148

[27] Grade, S. et al. Serum albumin reduces the antibacterial and cytotoxic effects of hydrogel-embedded colloidal silver nanoparticles. *RSC Adv. ***2**, 7190–7196 (2012).

[28] Haroun, A. A., Mashaly, H. M. & El-Sayed, N. H. Novel nanocomposites based on gelatin/HPET/chitosan with high performance acid red 150 dye adsorption. *Clean Technol. Environ. Policy ***15**, 367–374 (2013).

[29] Chen, X. & Schluesener. Nanosilver: a nanoproduct in medical application. *Toxicol. Lett. ***176,**1–12 (2008). 10.1016/j.toxlet.2007.10.004 18022772

[30] Varaprasad, K. et al. Hydrogel–silver nanoparticle composites: A new generation of antimicrobials. *J Appl. Polym. Sci. ***115**, 1199–1207 (2010).

[31] Mohan, Y. M., Lee, K., Premkumar, T. & Geckeler, K. E. Hydrogel networks as nanoreactors: A novel approach to silver nanoparticles for antibacterial applications. *Polymer. ***48**, 158–164 (2007).

[32] Ford, C. B., Funt, J. M., Abbey, D., Issi, L., Guiducci, C., Martinez, D. A., Delorey, T., Li, B. Y., White, T. C., Cuomo, C., RaoJudith, R. P., Berman, J., Thompson, D. A., Regev, T. A. The evolution of drug resistance in clinical isolates of *Candida albicans*. *eLife. ***4,** e00662 (2015). 10.7554/eLife.00662 PMC4383195 25646566

[33] Delaloye, J. & Calandra, T. Invasive candidiasis as a cause of sepsis in the critically ill patient. *Virulence. ***5**, 161–169 (2014). 24157707 10.4161/viru.26187 PMC3916370

[34] Anjum, S. & Abbasi, B. H. Biomimetic synthesis of antimicrobial silver nanoparticles using in vitro-propagated plantlets of a medicinally important endangered species: *Phlomis bracteosa*. *Int. J. Nanomed. ***11**, 1663–1675 (2016). 10.2147/IJN.S105532 PMC4853015 27217745

[35] Chudobova, D. et al. Complexes of silver(I) ions and silver phosphate nanoparticles with hyaluronic acid and/or chitosan as promising antimicrobial agents for vascular grafts. *Int. J. Mol. Sci. ***14**, 13592–13614 (2013). 23812079 10.3390/ijms140713592 PMC3742205

[36] Ibrahim, A. G., Sayed, A. Z., El-Wahab, H. A. & Sayah, M. M. Synthesis of poly(acrylamide-graft-chitosan) hydrogel: optimization of the grafting parameters and swelling studies. *Am. J. Polym. Sci. ***5,** 55–62 (2019).

[37] Muhammad, I., Saima, G., Murad, K. & Mi, K. Plant mediated green synthesis of anti-microbial silver nanoparticles. A review on recent trends. *Nanotechnol. Rev. ***5**, 119–135 (2016).

[38] Gupta, N., Vishal, H. & Shivakumar, G. Investigation of swelling behavior and mechanical properties of a pH-sensitive superporous hydrogel composite. *Iran. J. Pharm. Res. ***11**, 481–493 (2012). 24250471 PMC3832170

[39] Abd-Allah, W. M. & Fathy, R. M. Gamma irradiation effectuality on the antibacterial and bioactivity behavior of multicomponent borate glasses against methicillin-resistant staphylococcus aureus (MRSA). *J. Biol. Inorg. Chem. ***27**, 155–173 (2022). 35064832 10.1007/s00775-021-01918-z

[40] Rageh, M. M., El-Gebaly, R. H. & Afifi, M. M. Antitumor activity of silver nanoparticles in ehrlich carcinoma-bearing mice. *Naunyn Schmiedebergs Arch. Pharmacol. ***391**, 1421–1430 (2018). 30178417 10.1007/s00210-018-1558-5

[41] Wang, D., Yang, H., Zhou, Z., Chen, R. & Reed, S. H. XPF plays an indispensable role in relieving silver nanoparticle induced DNA damage stress in human cells. *Toxicol. Lett. ***288**, 44–54 (2018). 29462690 10.1016/j.toxlet.2018.02.022

[42] Zielinska, E., Zauszkiewicz-Pawlak, A., Wojcik, M. & Inkielewicz-Stepniak, I. silver nanoparticles of different sizes induce a mixed type of programmed cell death in human pancreatic ductal adenocarcinoma. *Oncotarget. ***9**, 4675–4697 (2018). 29435134 10.18632/oncotarget.22563 PMC5797005

[43] Kumar, D., Gihar, S., Shrivash, M. K., Kumar, P. & Kundu, P. P. A review on the synthesis of graft copolymers of chitosan and their potential applications. *Int. J. Biol. Macromol. ***163**, 2097–2112 (2022). 10.1016/j.ijbiomac.2020.09.060 32949625

[44] Samiullah, K. & Nazar, R. Effect of degree of cross-linking on swelling and on drug release of low viscous chit*osan/poly(vinyl a*lcohol) hydrogels. *Polym. Bull. ***71**, 2133–2158 (2014).

[45] Ma, J. et al. Ultraviolet-assisted synthesis of polyacrylamide-grafted chitosan nanoparticles and flocculation performance. *Carbohydr. Poly. ***15**, 565–575 (2016). 10.1016/j.carbpol.2016.06.002 27474601

[46] Zhang, Y., Xue, C., Xue, Y., Gao, R. & Zhang, X. Determination of the degree of deacetylation of chitin and chitosan by X-ray powder diffraction. *Carbohydr. Res. ***340**, 1914–1917 (2005). 15963961 10.1016/j.carres.2005.05.005

[47] Yang, Y. et al. One-step synthesis of amino-functionalized fluorescent carbon nanoparticles by hydrothermal carbonization of chitosan. *Chem. Commun. ***48**, 380–382 (2021). 10.1039/c1cc15678k 22080285

[48] Qian, X.-F., Yin, J., Feng, S., Liu, S.-H. & Zhu, Z.-K. Preparation and characterization of polyvinylpyrrolidone films containing silver sulfide nanoparticles. *J. Mater. Chem. ***11**, 2504–2506 (2001).

[49] Prashanth, K. V. H., Kittur, F. S. & Tharanathan, R. N. Solid state structure of chitosan prepared under different N-deacetylating conditions. *Carbohydr. Polym. ***50**, 27–33 (2002).

[50] Kong, L. et al. Preparation and characterization of nano-hydroxyapatite/chitosan composite scaffolds. *J. Biomed. Mater. Res. A ***75A**, 275–282 (2005). 10.1002/jbm.a.30414 16044404

[51] Govindan, S., Nivethaa, E. A. K., Saravanan, R., Narayanan, V. & Stephen, A. Synthesis and characterization of chitosan–silver nanocomposite. *Appl. Nanosci. ***2**, 299–303 (2012).

[52] Nityananda, A. & Kaushik, N. K. One pot synthesis of crystalline silver nanoparticles. *J. Nanomater. ***2**, 4–7 (2014).

[53] Raffi, M., Hussain, F., Bhatti, T.M., Akhter, J.J., Hameed, A., Hasan, M.M. Antibacterial characterization of silver nanoparticles against *E. coli* ATCC-15224. *J. Mater. Sci. Technol. ***24,** 192–196 (2008).

[54] Modoukpè, I. et al. Developments in research on the nutritional health-promoting properties of three traditional leafy vegetables commonly consumed in sub-Saharan Africa. *J. Herb. Med. ***40**, 100668 (2023).

[55] Kumar, S., Chaitanya, R. K., Preedy, V. R., *Chapter 20—Assessment of Antioxidant Potential of Dietary Components*, (eds. Preedy, V. R., Watson, R. R.) HIV/AIDS 239–253 (Academic Press, 2018).

[56] Granato, D. et al. Antioxidant activity, total phenolics and flavonoids contents: Should we ban in vitro screening methods?. *Food Chem. ***264**, 471–475 (2018). 29853403 10.1016/j.foodchem.2018.04.012

